# Alcohol Use and Subsequent Sex among HIV-Infected Patients in an Ethnic Minority Area of Yunnan Province, China

**DOI:** 10.1371/journal.pone.0061660

**Published:** 2013-04-23

**Authors:** Xiaofeng Luo, Song Duan, Qixiang Duan, Yongcheng Pu, Yuecheng Yang, Yingying Ding, Meiyang Gao, Na He

**Affiliations:** 1 Department of Epidemiology, School of Public Health, Fudan University, Shanghai, China; 2 The Key Laboratory of Public Health Safety (Fudan University), Ministry of Education, Shanghai, China; 3 Dehong Prefecture Center for Disease Control and Prevention, Mangshi, Yunnan Province, China; 4 Longchuan County Center for Disease Control and Prevention, Dehong Prefecture, Longchuan, Yunnan Province, China; University of Rochester, United States of America

## Abstract

**Objective:**

To examine alcohol use and subsequent HIV risky behaviors among a sample of predominately ethnic minority people living with HIV/AIDS (PLWHA) in a rural community in Yunnan Province, China.

**Method:**

A cross-sectional study with a face-to-face questionnaire interview was conducted among eligible participants.

**Results:**

In total, 455 (94.4%) out of 482 eligible HIV patients participated in the study. Of them, 82.6% were ethnic minorities; 15.4% were never married; 96.5% were sexually experienced; 55.4% had used drugs, 67% were receiving antiretroviral therapy (ART). Over 65% were ever drinkers; of whom 61.5% were current drinkers. Among current drinkers, 32.4% drank daily and 41.2% were hazardous drinkers. Chinese white wine was the preferred choice. Higher level of alcohol use among drinkers in the preceding month was positively associated with being males (OR = 2.76, 95%CI: 1.03–7.43), ethnic minorities (OR _Jingpo_ = 2.21, 95%CI: 1.06–4.59; OR _other minorities_ = 3.20, 95%CI: 1.34–7.62), higher education (OR_1–6_ = 1.98, 95%CI: 0.99–3.96; OR_≥7_ = 2.35, 95%CI: 1.09–5.06) and being ART-naive (OR = 2.69, 95%CI: 1.67–4.32). About 39% of ever drinkers reported having engaged in sex after drinking since HIV diagnosis. Those who were younger than 46 years (OR_16–25_ = 7.77, 95%CI: 1.22–49.60, OR_26–35_ = 2.79, 95%CI: 1.06–7.35, OR_36–45_ = 2.96, 95%CI: 1.57–7.58), hazardous drinkers (OR = 1.99, 95%CI: 1.00–3.97) and drug users (OR = 3.01, 95%CI: 1.19–7.58) were more likely to have had sex after drinking. Approximately 56% of drug users had used drugs after drinking.

**Conclusions:**

High prevalence of alcohol use and subsequent risky behaviors including sexual engagement and drug use among HIV patients in rural Yunnan require tremendous and integrated efforts for prevention and control of alcohol and drug abuse and HIV spreading.

## Introduction

In spite of substantial efforts aimed at reducing the spread of HIV, HIV incidence continues to remain at a high level throughout many parts of the world, with 2.5 million people being newly infected with HIV in 2011 only [1]. The dominant transmission mode was unprotected sexual intercourse which bridged HIV spreading between HIV-infected and non-infected individuals. Literature has showed that over 70% of people living with HIV/AIDS (PLWHA) remains sexually active after diagnosis with HIV infection [2], and that one-third of them engage in unprotected sexual behavior [3]. Some reports have shown the rates reached up to 84% [4].

Alcoholic beverages are widely consumed psychoactive substance throughout the world [5], and alcohol has long been recognized as a significant contributor to illness and injury, accounting for 4% of the global burden of disease [6]. Since the middle 1980s, the global literature suggests that alcohol consumption is associated with a number of sexual risk behaviors including premarital intercourse, multiple sexual partners, unprotected sex, sexual violence, as well as outcomes such as unwanted pregnancy, HIV/AIDS and other sexually transmitted infections (STIs) [7]–[9]. Moreover, heavy alcohol consumption tends to be more prevalent among PLWHA than among the general population [10]. In this context, alcohol-related problems and co-occurred unsafe sex further amplify considerable contributions of alcohol to the global burden of diseases [11].

Nevertheless, data on the association between alcohol use and sexual risk practice in China are limited, and mainly focused on specific sub-population such as drug users, migrants, female sex workers (FSWs) and men having sex with men (MSM) in metropolitan and coastal areas [12]–[15]. Little is known about alcohol use and its association with sexual risk practice among PLWHA in China. This is problematic as drinking is socially accepted and plays a significant role in daily life in China due to culture, custom, faith and belief [16], while sexual transmission replaced injection drug use (IDU) as the dominant mode of HIV transmission in China since 2007 [17] and 81.6% of the estimated 48,000 newly infected HIV patients in 2011 were due to unprotected sexual behaviors [18]. To fill this significant gap, we designed and conducted a community-based cross-sectional study in an ethnic minority area in Dehong Prefecture of Yunnan Province to examine prevalence and correlates of alcohol use and subsequent sexual engagement among HIV-infected patients. The study site was so chosen for the following concerns: 1) China's first HIV outbreak was observed among IDUs in 1989 in Dehong Prefecture of Yunnan Province which borders the drug-trafficking routes known as the “Golden Triangle” and has a high concentration of ethnic minorities [19]; 2) Yunnan Province has the largest number of reported HIV/AIDS cases among all Chinese provinces, accounting for 22% (93,567) of the total of 429,000 reported HIV/AIDS cases (PLWHs) in this country [20]; 3) ethnic minorities in this province have been disproportionally affected by the HIV epidemic [21]; and 4) sexual contact has replaced injection drug use (IDU) as the predominant mode of transmission in Yunnan Province since 2006 [19].

## Methods

### Ethics Statement

Written informed consent was obtained from all study participants. For participants aged 16 or 17 years, written informed consent was obtained from both the participants and their caretakers although 16 is the age of consent and marriage in the Dai and Jingpo culture. This study was approved by the Institutional Review Board (IRB) of Fudan University, Shanghai, China.

### Study Site and Participants

This study was conducted among reported HIV/AIDS cases in two towns of Dehong Prefecture, a Dai and Jingpo Autonomous Prefecture in the west of Yunnan Province that borders Myanmar. A roster of registered HIV/AIDS cases living in these two towns during May to August in 2012 was obtained from Dehong Prefecture Center for Disease Control and Prevention (CDC) as a sampling frame. All registered HIV/AIDS cases who aged 16 years or older and were able to provide informed consent were recruited for the study. For those aged 16 or 17 years, written informed consent was also obtained from their caretakers although 16 is the age of consent and marriage in the Dai and Jingpo cultures.

### Questionnaire Survey

A face-to-face, paper-pencil interview was administered by trained local public health workers in a separate room or private space (e.g., consulting room of clinic or the participant's home) that was designated by study participants. The primary variables of interest included demographic characteristics, alcohol use, drug use, sexual engagement and condom use after drinking, drug use after drinking and antiretroviral treatment (ART) status of the study participants. Participants were compensated with the equivalence of US $5 for their participation. All participants were assured of the confidentiality of their information on the informed consent form and throughout the interview.

### Measures

#### Alcohol Use

A drinker was defined as an individual who had drunk at least once a month for more than one year [22]. Two questions in the questionnaire were used to measure the prevalence of drinking: (1) “Have you ever drunk alcohol in your life?” and (2) “Did you drink alcohol in the preceding month?” If a respondent answered ‘yes’ to the first question, then this person was defined as an ever drinker. If a respondent answered “yes” to both questions, then this person was defined as a current drinker.

Current drinkers were asked about the types and quantities of beer and Chinese white wine or spirits consumed during the preceding month in order to measure alcohol (ethanol) consumption. We assumed an ethanol content of 4% for beer and 40% for Chinese white wine or spirits. The quantity (grams) of alcohol consumed was then calculated by multiplying the number of milliliters consumed by the percentage of ethanol contained in each type of beverage and 0.79 (conversion factors). If a drinker consumed both types of beverages, their absolute alcohol consumption was summed. We defined hazardous drinking as >14 drinks per week (196 gram of alcohol) for men aged 65 years or younger and >7 drinks (98 gram) per week for women of any age and for men older than 65 years [23], and moderate alcohol use was any alcohol use at less than hazardous levels [24].

Current drinker also provided the frequency of drinking during the preceding month (daily or nearly daily: at least 4 times per week; often: 1–3 times per week; occasionally: 1–3 times per month). Intoxicating and attending clinic due to drinking were also inquired.

#### Sexual Engagement after Drinking and Condom Use

Participants were asked sexual history, lifetime sexual experience after drinking with a HIV infection status, and frequency of condom use (never, sometimes, and always) for sex after drinking. Consistent use of condoms was defined in this study as using a condom every time during the sexual episodes after drinking.

#### Drug Use

A drug user was defined as an individual who had used illicit drug including opium, heroin, ephedrine, ketamine, methamphetamine or ecstasy at least once in his or her lifetime for nonmedical purposes. Behavior of drug use after drinking was also requested.

### Statistical Analysis

Data were analyzed using SPSS 17.0 for Windows (SPSS Inc., Chicago). In addition to descriptive analyses, tests of associations between two categorical variables were based on the chi-square test or Fisher's exact test, where appropriate. A multivariate ordinal logistic regression analysis adjusting for potential confounding variables was conducted to examine correlates of higher level of alcohol use among ever drinkers during the preceding month. Univariate and multivariate logistic regression analyses were also performed to explore correlates of subsequent sex after drinking among ever drinkers under HIV infection status. Respective odds ratio (OR) and 95% confidence interval (95%CI) were calculated. A significance level of 0.05 was used for all tests.

## Results

### Socio-demographic Characteristics

A total of 657 registered HIV/AIDS case, 175 (26.6%) were either migrating outside (114, or 17.3%) or incarcerated for drug use (61, or 9.3%) during the study period and thus were not identifiable or eligible for this study. Among the remaining 482 eligible HIV/AIDS cases, 27 (5.6%) did not participate in the study for refusal (24, or 5.0%) or illness (3, or 0.6%). Among the 455 participants, the mean age was 38.1 years old (SD = 8.8); 79 (17.4%) were Han (China's predominant ethnic group), 310 (68.1%) were of Jingpo minority, 62 (13.6%) were of Dai minority, and 4 (0.9%) were of other ethnic minorities; 17.8% were illiterate and 55.6% had only primary school education; 15.4% were never married; 67% were receiving ART. Male participants differed significantly from females in age, education, marital status and ART status (Table 1).

**Table 1:** Characteristics of study participants.

Variables	Male (n = 301)	Female (n = 154)	Total (N = 455)
	No. (%)	No. (%)	No. (%)
Current Age (years) (**χ^2^ = 17.23, ** ***p*** ** = 0.001**)			
16-	7(2.3)	16(10.4)	23(5.1)
26-	117(38.9)	55(35.7)	172(37.8)
36-	133(44.2)	53(34.4)	186(40.9)
≥46	44(14.6)	30(19.5)	74(16.3)
Ethnicity (χ^2^ = 0.14, *p* = 0.932)			
Han	52(17.3)	27(17.5)	79(17.4)
Jingpo	204(67.8)	106(68.4)	310(68.1)
others	45(15.0)	21(13.6)	66(14.5)
Education (years) **(χ^2^ = 99.43, ** ***p*** ** = 0.002)**			
0 (Illiterate)	42(14.0)	39(25.3)	81(17.8)
1–6	178(59.1)	75(48.7)	253(55.6)
≥7	81(26.9)	40(26.0)	121(26.6)
Marital Status (**χ^2^ = 23.23, ** ***p*** **<0.001**)			
Never married	62(20.6)	8(5.2)	70(15.4)
First married	149(49.5)	76(49.4)	225(49.5)
Remarried	44(14.6)	38(24.7)	82(18.0)
Divorced or widowed	46(15.3)	32(20.8)	78(17.1)
Ever Used Drugs (**χ^2^ = 249.75, ** ***p*** **<0.001**)			
Yes	246(81.7)	6(3.9)	252(55.4)
No	55(18.3)	148(96.1)	203(44.6)
On ART Currently (**χ^2^ = 7.67, ** ***p*** ** = 0.006**)			
Yes	190(63.1)	117(76.0)	307(67.5)
No	111(36.9)	37(24.0)	148(32.5)
Sexually Experienced^a^ (χ^2^ = 0.59, *p* = 0.443)			
Yes	288(96.0)	150(97.4)	438(96.5)
No	12(4.0)	4(2.6)	16(3.5)

a : one participants refused to provide any sexual information.

### Alcohol Use: Lifetime and in the Preceding Month

Table 2 presents detailed information about alcohol use among study participants. Over 65.1% (296/455) were defined as ever drinkers, of whom 56.1% started drinking before age 18 and 61.5% were current drinkers (i.e., drank in the preceding month). In addition, 20.6% ever drinkers regarded drinking as having no harm to the disease status of HIV/AIDS. Among current drinkers, 32.4% drank daily; 52.7% drank Chinese white wine only; 20.3% drank beer only, 25.3% were intoxicated at least once during the preceding month, 2.9% male respondents attended clinic due to drinking in the preceding month. Compared to females, males were more likely to be ever drinkers, to start drinking at an earlier age, to have a higher frequency of drinking, to drink Chinese white wine and to consume more alcohol in the preceding month (Table 2).

**Table 2:** Alcohol use among study participants.

Variables	Male (n = 301)	Female (n = 154)	Total (N = 455)
	No. (%)	No. (%)	No. (%)
Ever Drinker (**χ^2^ = 237.62, ** ***p*** **<0.001**)			
Yes	270(89.7)	26(16.9)	296(65.1)
No	31(10.3)	128(83.1)	159(34.9)
Age at Drinking Initiation among Ever Drinkers (years) **(χ^2^ = 9.84, ** ***p*** ** = 0.002)**			
≤18	159(58.9)	7(26.9)	166(56.1)
≥19	111(41.1)	19(73.1)	130(43.9)
Drinking Does Harm to Disease Status of HIV/AIDS (χ^2^ = 2.91, p = 0.088)			
Yes	211(78.1)	24(92.3)	235(79.4)
No	59(21.9)	2(7.7)	61(20.6)
Frequency of Drinking in the Preceding Month among Ever Drinkers (**χ^2^ = 8.87, ** ***p*** ** = 0.031**)			
Daily or nearly daily	59(21.9)	0(0)	59(19.9)
Often	42(15.6)	3(11.5)	45(15.2)
Occasionally	70(25.9)	8(30.8)	78(26.4)
Never	99(36.7)	15(57.7)	114(38.5)
Type of Alcoholic Beverage Consumed in the Preceding Month among Current Drinker (**χ^2^ = 13.62, ** ***p*** ** = 0.001**)			
Chinese white wine only	93(54.4)	3(27.3)	96(52.7)
Beer only	30(17.5)	7(63.6)	37(20.3)
Both	48(28.1)	1(9.1)	49(26.9)
Level of Alcohol Use in the Preceding Month among Ever Drinkers(gram) (χ^2^ = 5.09, *p* = 0.078)			
None	99(36.7)	15(57.7)	114(38.5)
Moderate level	99(36.7)	8(30.8)	107(36.1)
Hazardous level	72(26.7)	3(11.5)	75(25.3)
Intoxicated in the Preceding Month among Current Drinkers (*p* a = 0.875)			
Yes	43(25.1)	3(27.3)	46(25.3)
No	128(74.9)	8(72.7)	136(74.7)
Attended Clinic Due to Drinking in the Preceding Month among Current Drinkers			
Yes	5(2.9)	0(0)	5(2.7)
No	166(97.1)	11(100)	177(97.3)
Ever Used Drug after Drinking (*p* a = 0.090)			
Yes	140(56.9)	1(16.7)	141(56.0)
No	106(43.1)	5(83.3)	111(44.0)
Sex after Drinking under HIV Infection Status among Ever Drinkers (**χ^2^ = 9.41, ** ***p*** ** = 0.001**)			
Yes	109(42.4)	3(11.5)	112(39.6)
No	148(57.6)	23(88.5)	171(60.4)
Condom Use during Sex after Drinking under HIV Infection Status (**χ^2^ = 203.42, ** ***p*** **<0.001**)			
Never	48(44.0)	2(66.7)	50 (44.6)
Sometimes	39(35.8)	0(0)	39(34.8)
Always	22(20.2)	1(33.3)	23 (20.5)

a Fisher's exact test.

### Prevalence and Correlates of Higher Levels of Alcohol Use in the Preceding Month

Among the 296 ever drinkers, 36.1% were categorized as moderate level users, and 25.3% as hazardous level users in the preceding month. Multivariate ordinal logistic regression analysis indicated that after controlling for potential confounding variables including current age, sex, ethnicity, education, marital status, age at drinking initiation, ever used drugs and current status of ART, higher level of alcohol use among ever drinkers in the preceding month was positively associated with being males (OR = 2.76, 95%CI: 1.03–7.43), being ethnic minorities (OR _Jingpo_ = 2.21, 95%CI: 1.06–4.59; OR _other minorities_ = 3.20, 95%CI: 1.34–7.62), having received higher education(OR_1–6_ = 1.98, 95%CI: 0.99–3.96; OR_≥7_ = 2.35, 95%CI: 1.09–5.06) and not receiving ART (OR = 2.69, 95%CI: 1.67–4.32), but not significantly associated with age, marital status, age at drinking initiation and drug use (Table 3).

**Table 3:** Prevalence and correlates of higher level of alcohol use in the preceding month among ever drinkers (n = 296).

Variables	None	Moderate level	Hazardous Level	OR(95%CI)^a^^, ^ b	*p* b
	No. (%)*	No. (%)*	No. (%)*		
Current Age (years)					
16-	4(44.4)	3(33.3)	2(22.2)	1.00	
26-	47(38.5)	42(34.4)	33(27.0)	1.88(0.48–7.38)	0.368
36-	40(33.3)	48(40.0)	32(26.7)	2.31(0.58–9.24)	0.236
≥46	23(51.1)	14(31.1)	8(17.8)	1.59(0.36–7.08)	0.544
Sex					
Male	99(36.7)	99(36.7)	72(26.7)	2.76(1.03–7.43)	**0.044**
Female	15(57.7)	8(30.8)	3(11.5)	1.00	
Ethnicity					
Han	19(43.2)	20(45.5)	5(11.4)	1.00	
Jingpo	87(41.2)	65(30.8)	59(28.0)	2.21(1.06–4.59)	**0.034**
Others	8(19.5)	22(53.7)	11(26.8)	3.20(1.34–7.62)	**0.009**
Education (years)					
0 (Illiterate)	25(56.8)	13(29.5)	6(13.6)	1.00	
1–6	62(37.1)	57(34.1)	48(28.7)	1.98(0.99–3.96)	0.053
≥7	27(31.8)	37(43.5)	21(24.7)	2.35(1.09–5.06)	**0.030**
Marital Status					
Never married	27(45.0)	17(28.3)	16(26.7)	0.83(0.39–1.76)	0.628
First married	54(37.0)	56(38.4)	36(24.7)	1.35(0.71–2.57)	0.363
Remarried	15(34.9)	15(34.9)	13(30.2)	1.58(0.70–3.57)	0.269
Divorced or widowed	18(38.3)	19(40.4)	10(21.3)	1.00	
Age at Drinking Initiation (years)					
≤18	64(38.6)	60(36.1)	42(25.3)	0.98(0.62–1.54)	0.922
≥19	50(38.5)	61(46.9)	19(14.6)	1.00	
Ever Used Drugs					
Yes	90(39.1)	77(33.5)	63(27.4)	0.72(0.36–1.45)	0.360
No	24(36.4)	30(45.5)	12(18.2)	1.00	
On ART Currently					
Yes	86(46.2)	66(35.5)	34(18.3)	1.00	
No	28(25.5)	41(37.3)	41(37.3)	2.69(1.67–4.32)	**<0.001**

a OR: Odds Ratio, CI: Confidence Interval;

b Obtained from multivariate ordinal logistic regression analysis adjusting for potential confounding variables listed in the table;

* Proportions were calculated in the row.

### Drug Use

Roughly a half (55.4%) of participants were drug users, and drug use was much more common among male patients than females (81.7% vs. 3.9%, χ^2^ = 249.75, p<0.001) (Table 1). About 56.0% (141/256) drug users had used drugs after drinking (Table 2).

### Sexual Engagement after Drinking under HIV Infection Status

Among the 455 participants, one refused to provide any sex related information. Of the rest, 438 (96.5%) were sexual experienced (Table 1). Among the 296 ever drinkers, 283 (95.6%) had sexual experience. Of them, 112 (39.6%) had engaged in sex after drinking under HIV infection status, 50 (44.6%) of whom had never used condoms and only 23 (8.1%) had consistently used condoms for sex after drinking (Table 2).

Univariate logistic regression analyses showed that sexual engagement after drinking under HIV infection status was significantly correlated with age, sex, ethnicity, level of alcohol use in the preceding month and drug use. Multivariate logistic regression analysis indicated that after adjusting for potential confounding variables including current age, sex, ethnicity, education, marital status, age at drinking initiation, level of alcohol use in the preceding month, ever used drugs and current status of ART, those who were younger than 46 years (OR_16–25_ = 7.77, 95%CI: 1.22–49.60, OR_26–35_ = 2.79, 95%CI: 1.06–7.35, OR_36–45_ = 2.96, 95%CI: 1.57–7.58), were hazardous drinkers (OR = 1.99, 95%CI: 1.00–3.97) and drug users (OR = 3.01, 95%CI: 1.19–7.58) were more likely to have sex after drinking under HIV infection status(Table 4).

**Table 4:** Prevalence and correlates of subsequent sex after drinking under HIV infection status among ever drinkers (n = 283).

Variables	Prevalence of subsequent sex after drinking (%)	Univariate		Multivariate	
		OR(95%CI)^a^	*p*	OR(95%CI)	*p*
Current Age (years)					
16-	50.0	3.40(0.72–16.10)	0.123	7.77(1.22–49.60)	**0.030**
26-	40.9	2.35(1.06–5.22)	**0.036**	2.79(1.06–7.35)	**0.038**
36-	44.0	2.67(1.21–5.91)	**0.016**	2.96(1.57–7.58)	**0.024**
≥46	22.7	1.00		1.00	
Sex					
Male	42.4	5.65(1.65–19.29)	**0.006**	3.40(0.80–14.51)	0.098
Female	11.5	1.00		1.00	
Ethnicity					
Han	19.0	1.00		1.00	
Jingpo	42.8	3.18(1.40–7.21)	**0.006**	1.82(0.70–4.71)	0.220
Others	45.0	3.48(1.29–9.36)	**0.014**	2.44(0.81–7.36)	0.115
Education (years)					
0 (Illiterate)	51.2	1.99(0.93–4.27)	0.078	2.38(0.95–5.95)	0.065
1–6	39.1	1.22(0.70–2.12)	0.490	0.88(0.47–1.65)	0.689
≥7	34.6	1.00		1.00	
Marital Status					
Never married	47.9	1.57(0.69–3.58)	0.284	1.11(0.45–2.74)	0.826
First married	38.4	1.06(0.54–2.11)	0.865	1.05(0.49–2.25)	0.893
Remarried	37.2	1.01(0.43–2.39)	0.980	1.13(0.42–3.03)	0.805
Divorced or widowed	37.0	1.00		1.00	
Age at Drinking Initiation (years)					
≤18	40.1	1.05(0.65–1.70)	0.832	0.83(0.48–1.45)	0.518
≥19	38.9	1.00		1.00	
Level of Alcohol Use in the Preceding Month					
None	36.9	1.00		1.00	
Moderate level	31.6	0.84(0.48–1.48)	0.550	0.89(0.46–1.70)	0.715
Hazardous level	60.3	1.91(1.05–3.49)	**0.035**	1.99(1.00–3.97)	**0.050**
Ever Used Drugs					
Yes	47.0	4.97(2.41–10.24)	**<0.001**	3.01(1.19–7.58)	**0.019**
No	15.2	1.00		1.00	
On ART Currently					
Yes	26.1	0.87(0.56–1.37)	0.555	1.48(0.83–2.65)	0.188
No	28.8	1.00		1.00	

a OR: Odds Ratio, CI: Confidence Interval, obtained from binary logistic regression analysis.

## Discussion

This study, to the best of our knowledge, is the first community-based study specifically designed to examine alcohol use and subsequent sex after alcohol use among HIV-infected patients in ethnic minority area in China that have been highly affected by the HIV epidemic. We observed that 40.1% of HIV-infected patients reported having had alcohol drinking in the preceding month. This proportion is similar to that (43%) among clinically observed HIV-infected patients [25], and lower than that (53%) among a population-based sample in the United States [26]. The proportion (41.2%) of having hazardous alcohol use among current drinkers of our study participants was higher than that (36.7% and 15% respectively) of HIV-infected patients in the United States [25], [26]. Furthermore, the present study indicated that homemade Chinese white wine was the primary choice of HIV-infected patients for alcohol drinking in the study areas and 2.5% of current drinkers had attended clinics due to drinking at least once during the preceding month. Homemade Chinese white wine might be associated with an increased risk of harm because of a high concentration of ethanol and unknown but potentially dangerous impurities or contaminants in these beverages [27]. Moreover, about 20.6% of the study participants did not realize the harmful effects of alcohol drinking. Matthew et al. had found that hazardous drinking and alcohol abuse and dependence were associated with a higher prevalence of CVD (cardiovascular disease) compared with infrequent and moderate drinking among HIV infected men [28], and many other studies suggest an association between alcohol use and decreased adherence to ART and poor HIV treatment outcomes among HIV patients [10], [29], [30]. Given the numerous adverse or harmful physical and psychological effects of alcohol use to HIV-infected patients, the high prevalence of alcohol use in general and heavy alcohol use in particular among HIV-infected patients in our study underscores the urgent need of health education and intervention programs targeted at reducing alcohol use and abuse among HIV-infected patients in the study area.

Consistent with observations on the general population [31], [32], HIV-infected males in this study were at significantly higher risk of high level of alcohol use than HIV-infected females. Meanwhile, HIV-infected patients in the study who were ethnic minorities were more likely to be engaged in high level of alcohol use than those who were the Han majority. Li et al argue that ethnic minorities in China have a strong tradition of drinking, especially during specific festivals, and tend to exhibit a higher prevalence of alcohol abuse and disorders than the Han majority [33]. HIV-infected patients who had received higher education were also shown to be at significantly higher risk of high level of alcohol use. This was probably due to the fact that those at higher education level might be at higher social status and with higher income so as to have more opportunities to participate in social events where high level of alcohol use was very likely to occur, or to have more money to pay for the expenditure of drinking. Furthermore, ART-naive HIV-infected patients in this study consumed more alcohol than those under ART. There are at least two reasons to explain this. First, at each follow-up visit for prescription of antiretroviral drugs (ARV) and monitoring of disease progression, HIV-infected patients receiving ART had been reiterated the importance of adherence to the treatment and regimens as well as the importance of avoiding alcohol use and abuse; Second, it was also likely that ART-naive patients perceived that they were at relatively good condition of disease status, and therefore were unaware of the importance of minimizing alcohol consumption. Taken together the high prevalence and the significant correlates of heavy alcohol consumption among HIV-infected patients, future prevention and intervention programs for alcohol control among HIV-infected patients should pay more attentions to those who are males, ethnic minorities, with high education level and not with ART.

A substantial literature including the limited studies in China suggested that alcohol use and abuse are positively associated with a number of risky sexual behaviors and outcomes [7]–[9], [12]–[15]. However, no such studies have been done among HIV-infected patients in China. In the present study, nearly 40% of HIV-infected patients who were ever drinkers reported having had sex after drinking since their HIV diagnosis, with significantly higher proportions observed among the younger ones, hazardous drinkers and drug users. Younger people are generally active in sex and thus they are likely to engage in sexual practice upon the effect of alcohol. More alcohol consumption would increase the individuals' risk engaging in unsafe sexual behavior. Stein et al. had found hazardous drinkers were more than five times likely to report unprotected sex and have multiple partners among HIV-infected persons [34]. Although diminished libido and impaired sexual performance are common sequelae of chronic drug use, alcohol affects sexual arousal via pharmacological (content) and psychological (expectancy) [9]. Thus, drug users tended to have more sex after alcohol drinking. More importantly, only 8.1% of the study participants who were ever drinkers reported having always used condoms during sex after drinking. This was probably due to that restricted cognitive capacity after drinking made one to focus on immediate sexual arousal rather than the risk of HIV/STI when negotiating condom use [35]–[37], and that having a “risky” personality exposed HIV-infected patients to engaging in both drinking and risky sex [38], [39]. The high prevalence of sex after drinking and the low rate of condom use among HIV-infected patients in the study area imply the great potential of secondary HIV transmission or spreading from them to their sexual partners, underscoring the urgency and importance of implementation of alcohol control programs targeting HIV-infected patients and their sexual partners, with more efforts to younger patients and those who are using drugs.

More than a half (55.4%) of the study participants was ever drug users. This was not surprise since injection drug use was the predominant HIV transmission mode through middle 2000 s in Dehong Prefecture [40] and has come to play an equally significant role to sexual contact in HIV transmission since 2006. Nevertheless, about 56.0% of the drug users reported having used drugs after alcohol drinking. The high prevalence of drug use, the high prevalence of alcohol use and risky engagement in drug use and/or sex after alcohol drinking among HIV-infected patients suggest a great potential of secondary HIV transmission in this area, calling for urgent and tremendous prevention and intervention efforts for the dual epidemics of substance abuse and HIV, especially among HIV-infected patients and their partners.

This study has several limitations. First, since this was a cross-sectional study causal inferences cannot be made. Second, alcohol use and sexual behaviors are sensitive personal topics, thus any self-reported measures are subject to recall bias including deliberate concealment. Third, it might have been more suitable to examine occurrence and correlates of ‘risky sexual behavior’ (e.g., sexual intercourse without condom) instead of ‘sex behavior’ after alcohol use. However, in the present study, we were more interested in examining occurrence and correlates of sex after alcohol use in general rather than the ‘risky or unprotected sex (i.e., sex without condom use) in particular among ever drinking HIV/AIDS patients due to concerns that alcohol generally restricted one's cognitive capacity and consequently made one decrease condom use and increase the opportunity of HIV transmission [35]–[37], and that some of the study participants (i.e., HIV/AIDS patients) might have provided ‘socially acceptable’ answers by reporting that they had always used condoms for sex under the influence of alcohol use. In fact, very few (8.1%) of the study participants who were ever drinkers reported having always used condoms during sex after drinking. Finally, we did not test for other STIs such as syphilis and herpes simplex virus-2 that have been found to be prevalent in Chinese HIV patients [41]. Future studies should be extended to examine these STIs as outcomes of sex after alcohol use.

Nonetheless, findings from this study have important implications for future harm reduction programs targeting alcohol use and drug use as well as HIV prevention programs in rural Yunnan, and particularly among HIV-infected ethnic minority males. First, there is an urgent need to implement health education and intervention programs to prevent alcohol abuse in rural Yunnan, especially among HIV patients. Second, use and misuse of alcohol and illicit drugs have become a severe social and public health challenge that requires tremendous and integrated efforts in research and control. Third, given that Yunnan is one of China's HIV epicenters, the high prevalence of sex after alcohol use and the low rate of condom use during sex underscore the importance of enhanced condom promotion programs and empowerment programs for women especially partners or spouses of HIV-infected males to negotiate condom use. Finally, this study also mirrors a clue for further studies examining the association between alcohol use and adherence to ART.
